# Prototyping a Secure and Usable User Authentication Mechanism for Mobile Passenger ID Devices for Land/Sea Border Control

**DOI:** 10.3390/s24165193

**Published:** 2024-08-11

**Authors:** Maria Papaioannou, Georgios Zachos, Georgios Mantas, Emmanouil Panaousis, Jonathan Rodriguez

**Affiliations:** 1 Instituto de Telecomunicações, 3810-193 Aveiro, Portugal; g.zachos@av.it.pt (G.Z.); gimantas@av.it.pt (G.M.); jonathan@av.it.pt (J.R.); 2 Faculty of Engineering and Science, University of Greenwich, Chatham Maritime ME4 4TB, UK; e.panaousis@greenwich.ac.uk; 3 Faculty of Computing, Engineering and Science, University of South Wales, Pontypridd CF37 1DL, UK

**Keywords:** adaptive user authentication, risk-based user authentication, risk estimation, novelty detection, outlier detection, mobile passenger ID devices, prototype development

## Abstract

As the number of European Union (EU) visitors grows, implementing novel border control solutions, such as mobile devices for passenger identification for land and sea border control, becomes paramount to ensure the convenience and safety of passengers and officers. However, these devices, handling sensitive personal data, become attractive targets for malicious actors seeking to misuse or steal such data. Therefore, to increase the level of security of such devices without interrupting border control activities, robust user authentication mechanisms are essential. Toward this direction, we propose a risk-based adaptive user authentication mechanism for mobile passenger identification devices for land and sea border control, aiming to enhance device security without hindering usability. In this work, we present a comprehensive assessment of novelty and outlier detection algorithms and discern OneClassSVM, Local Outlier Factor (LOF), and Bayesian_GaussianMixtureModel (B_GMM) novelty detection algorithms as the most effective ones for risk estimation in the proposed mechanism. Furthermore, in this work, we develop the proposed risk-based adaptive user authentication mechanism as an application on a Raspberry Pi 4 Model B device (i.e., playing the role of the mobile device for passenger identification), where we evaluate the detection performance of the three best performing novelty detection algorithms (i.e., OneClassSVM, LOF, and B_GMM), with B_GMM surpassing the others in performance when deployed on the Raspberry Pi 4 device. Finally, we evaluate the risk estimation overhead of the proposed mechanism when the best performing B_GMM novelty detection algorithm is used for risk estimation, indicating efficient operation with minimal additional latency.

## 1. Introduction

Industries such as transportation increasingly rely on digital technologies to improve efficiency and infrastructure, promoting economic and social cohesion within the European Union (EU) [1,2]. For instance, the growing influx of visitors to the EU necessitates innovative border control solutions, such as mobile devices for passenger identification at land and sea borders, ensuring convenience and safety for both passengers and officers [1]. These devices, however, laden with sensitive personal data, attract malicious actors [3,4]. Therefore, robust user authentication mechanisms are crucial to secure these devices and protect sensitive data [1,3,4,5,6].

Public safety mobile authentication approaches have been explored to secure such devices. According to NIST Special Publication 8080 [7], most of the existing user authentication approaches are infeasible for public safety use in the field due to their inconvenience for first responders [8]. Subsequently, it is critical to research, design, and implement novel secure and usable user authentication mechanisms that will increase the level of device security of the upcoming passenger identification mobile devices while ensuring that border control officers will be able to complete their missions in an effective and efficient manner [7].

However, there is often a perceived trade-off between security and usability [9,10]. In particular, increased security measures often introduce complexity that can hinder usability, leading to frustration and non-compliance [11]. In addition, users may prioritize convenience over security, undermining security protocols, such as reusing passwords or writing them down [12], or bypass restrictive measures, increasing risks [13,14]. Finally yet importantly, the integration of security into the development process is often overlooked, leading to a lack of usability in security features. Early inclusion of usability requirements in the design and development stages can mitigate this issue [11].

To address this challenge, risk-based and adaptive user authentication approaches have been proposed, aiming to enhance security without hindering usability [6,9,10]. Enhancing user authentication with risk-based and adaptive user authentication methods offers two main benefits. Firstly, it increases efficiency by triggering re-authentication only when necessary, thus conserving resources. Secondly, it adjusts re-authentication levels adaptively based on real-time risk assessments [15,16]. For example, a low risk score allows the user to remain signed in, a medium score requires uni-modal authentication (e.g., keystroke dynamics), and a high score necessitates multi-modal authentication (e.g., keystroke dynamics and voice) or other countermeasures like device locking [15,17]. Therefore, it is clear that accurate risk estimation (i.e., computation of an accurate risk score of an action or event) plays a key role in risk-based adaptive user authentication as it might impact its overall usability and security [17,18].

In principle, the different approaches proposed over the years for risk estimation can be qualitative or quantitative [19]. Qualitative methods, though widely used, rely heavily on expert judgment, making them subjective and less suitable for real-world security solutions [20,21]. In contrast, quantitative approaches, which are emerging as a more reliable alternative, aim to improve accuracy and reliability in risk-based user authentication [22]. However, there remains a need for more effective quantitative methods tailored specifically for continuous user authentication on smartphones [22].

In our previous work in [6], we proposed, for the first time, a novel risk-based adaptive user authentication mechanism to address this challenge. Afterwards, we tested and evaluated a set of popular classification algorithms for risk-based authentication, including the Naïve Bayes [23], the Support Vector Machine [24], the k-Nearest Neighbor [25], and the Decision Tree [25] algorithms on the HuMIdb (Human Mobile Interaction database) dataset to discern the most effective ones for the proposed mechanism [6,9]. The evaluation results indicated impact of overfitting (i.e., accuracy: 100%) and thus, we considered novelty detection algorithms to overcome this challenge and demonstrate high performance [6]. To the best of our knowledge, this was the first time that novelty detection algorithms had been considered for risk-based adaptive user authentication demonstrating promising results (i.e., LOF 97%, KNN_average 99%, and OneClassSVM 95%) [6].

Extending our work in [6], we evaluated additional novelty detection algorithms such as Gaussian Kernel Density Estimation (G_KDE), Deep One-Class Classification (DeepSVDD), Bayesian Gaussian Mixture Model (B_GMM), and Parzen Window Kernel Density Estimation (PW_KDE), and presented the evaluation results in our work in [26]. The evaluation demonstrated exceptional performance for G_KDE, PW_KDE, and B_GMM novelty detection algorithms. In further investigation, in [27], we also explored the concept of outlier detection for the proposed mechanism in [6]. Specifically, four outlier detection algorithms—Isolation Forest (IF), Minimum Covariance Determinant (MCD), AutoEncoder, and KNN-based Outlier Detection—were evaluated, with MCD emerging as the most accurate and effective in identifying outliers within the dataset [27].

Therefore, in this paper, we extend our previous works by the following directions:

- Initially, a comprehensive overall assessment across all novelty and outlier detection algorithms, presented in [6,26,27], is conducted, using the HuMIdb dataset [28,29] to identify the most effective ones for integration into the proposed risk-based adaptive user authentication mechanism. According to our findings, the three novelty detection algorithms, namely OneClassSVM, LOF, and B_GMM, demonstrated a balance of high performance and likely generalizability to new data, outperforming the rest of the novelty detection and outlier detection algorithms.
- Additionally, as a second extension of our previous works, we developed the proposed mechanism as an application on a Raspberry Pi 4 Model B device (i.e., playing the role of the mobile device for passenger identification for land and sea border control).
- In addition, as third extension, we developed the three best performing novelty detection algorithms (i.e., OneClassSVM, LOF, and B_GMM), integrated them into the risk-based adaptive user authentication mechanism, and evaluated their detection performance. The evaluation results demonstrated that B_GMM surpasses the others (i.e., OneClassSVM, LOF) in performance when deployed on the Raspberry Pi 4 device.
- Finally, as a fourth extension of our previous works, we evaluated the risk estimation overhead of the developed mechanism, when the best performing B_GMM novelty detection algorithm was used for risk estimation, indicating efficient operation with minimal additional latency.

The rest of the paper is structured as follows: Section 2 gives an overview of the proposed risk-based adaptive user authentication mechanism, describing the proposed mechanism’s core components. Section 3 covers the comprehensive overall assessment of all novelty and outlier detection algorithms to identify the most effective ones for integration into the proposed mechanism. Section 4 presents the functional description of the proposed mechanism, while Section 5 provides details on its prototype implementation. Section 6 presents the performance evaluation of the proposed mechanism running on a Raspberry Pi 4 Model B device. Finally, Section 7 concludes the paper.

## 2. Overview of the Proposed Risk-Based Adaptive User Authentication Mechanism

### 2.1. Mechanism Components

**Figure 1:**
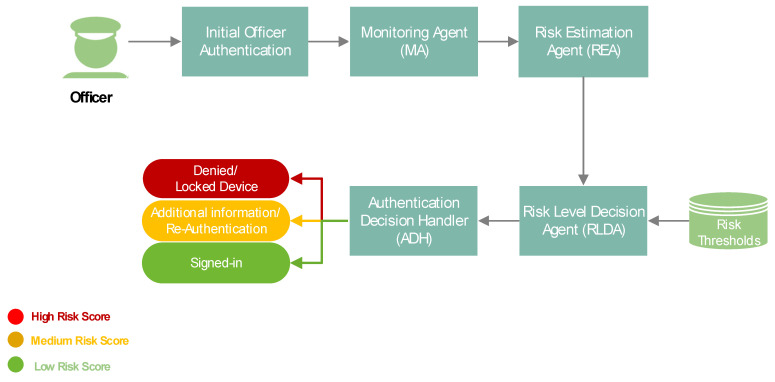
The high-level overview of the components of the proposed risk-based adaptive user authentication mechanism.

The core components of the proposed risk-based adaptive user authentication mechanism, also depicted in Figure 1, include the following:

#### 2.1.1. Initial Officer Authentication

An authentication process based on something the Officer knows (i.e., username and password) is initiated. In case that the validity of the claimed identity of the Officer requesting access to the device is not verified (e.g., due to interruptions in the Officer’s activity in the initial Officer authentication), the initial authentication is considered unsuccessful, and the Officer is denied access to the mobile device. On the contrary, when the claimed identity of the Officer requesting access to the device is verified, the initial authentication is considered successful. After successful initial Officer authentication, the Officer gains access to the mobile device and the Monitoring Agent (MA) starts running in the background without interrupting the Officer’s activity.

#### 2.1.2. Monitoring Agent (MA)

This component operates silently in the background, ensuring that monitoring does not interrupt the use of the device, and thus not impeding the Officer’s daily activities and missions. The MA component is responsible for collecting data related to the Officer’s activity and context. After specific predefined times, the collected data are compiled into a dataset, which is then sent to the Risk Estimation Agent (REA) component.

#### 2.1.3. Risk Estimation Agent (REA)

This component receives the dataset from the MA component and performs feature normalization on it to ensure that all input features contribute equally to the subsequent analysis. Afterwards, REA applies a detection algorithm to the normalized data to classify each entry as legitimate or malicious. In particular, REA applies one of the most efficient detection algorithms as identified from the analysis in Section 3 below. Based on the detection algorithm’s output and a predefined risk estimation formula, REA computes a real-time risk score. This score assesses the likelihood of a malicious activity (e.g., a malicious actor has taken over the device) based on deviations from expected activity and context of a legitimate user. The risk score is dynamically updated as new data are collected and forwarded to the Risk Level Decision Agent (RLDA) component.

#### 2.1.4. Risk Level Decision Agent (RLDA)

This component receives the computed risk score from REA and compares it against predefined risk thresholds to classify the risk score as low, medium, or high. Afterwards, RLDA communicates the risk level decision to the Authentication Decision Handler (ADH) component.

#### 2.1.5. Authentication Decision Handler (ADH)

This component receives the risk level decision from the RLDA component and adapts to appropriate authentication requirements accordingly, from seamless access at low risk to additional authentication steps (i.e., re-authentication) if the risk score is medium, or device lock if the risk score is high.

## 3. Novelty vs. Outlier Detection for Risk Estimation in the Proposed Mechanism

In [6], we examined the effectiveness of renowned classification algorithms for risk-based authentication, including the Naïve Bayes [23], the Support Vector Machine [24], the k-Nearest Neighbor [25], and the Decision Tree [25] algorithms. We trained and tested these algorithms over the HuMIdb dataset, which, to the best of our knowledge, is the most recent publicly available dataset for behavioral user authentication [28,29]. The performance of the classification algorithms was assessed using metrics such as accuracy, precision, recall, and F1 score. Due to the observed overfitting issues (i.e., the generated models used to become very closely related to training data with specific training features such us pressure, and thus the models achieved perfect accuracy scores), we explored the concept of novelty detection for the proposed mechanism in [6]. Therefore, we trained and tested the following novelty detection algorithms, noting their superior efficacy: one-class SVM, Local Outlier Factor (LOF), and an optimized KNN for novelty detection. It bore mentioning that the novelty detection algorithms were trained and tested over the same HuMIdb dataset as the classification algorithms. The optimized KNN algorithm stood out with an accuracy of 99%, closely followed by LOF and OneClassSVM with accuracies of 97% and 95%, respectively. In terms of other evaluation metrics such as precision, recall, and F1 score, KNN slightly outperformed both LOF and OneClassSVM, maintaining its edge in overall performance. Thus, applying novelty detection algorithms for risk estimation demonstrated a novel approach for risk estimation in risk-based adaptive user authentication.

Following these promising results, in [26], we investigated further the concept of novelty detection for risk-based user authentication, and thus we aimed to test more novelty detection algorithms and evaluate to identify the most appropriate ones that can be applied to the proposed mechanism. In particular, expanding upon our prior work in [6], we evaluated the following novelty detection algorithms: (i) Deep One-Class Classification (DeepSVDD) [30,31], (ii) Gaussian Kernel Density Estimation (G_KDE) [32], (iii) Parzen Window Kernel Density Estimation (PW_KDE) [32], and (iv) Bayesian Gaussian Mixture Model (B_GMM) [23,33]. These algorithms were chosen for their prominence in novel detection approaches, as they are widely considered in the literature to be suitable detection algorithms for behavioral biometric-based authentication [26,32,34,35,36,37,38]. Based on our findings [26], for the HuMIdb dataset, three of these novelty detection algorithms—G_KDE, PW_KDE, and B_GMM—showed exceptional performance, achieving near-perfect accuracy rates of 99%. These algorithms outperformed the DeepSVDD, which scored 86% in accuracy. Additionally, in terms of precision, recall, and F1 score, G_KDE, PW_KDE, and B_GMM consistently surpassed the DeepSVDD algorithm, underlining their superior capability in handling novelty detection tasks within this dataset.

Furthermore, in our more recent work [27], we investigated the concept of outlier detection for risk-based user authentication. Outlier detection algorithms are advantageous because they require only a minimal dataset from the abnormal class, making them ideal for scenarios with scarce abnormal samples [17,27,39]. Thus, we trained and tested the following four outlier detection algorithms: Isolation Forest (IF) [34], Minimum Covariance Determinant (MCD) [34], AutoEncoder [40], and KNN-based Outlier Detection [27]. Within the evaluation of the four outlier detection algorithms, the MCD emerges as the most accurate, achieving a notable 97% accuracy rate. MCD also leads in other key metrics, boasting the highest recall and F1-score at 97% and 98%, respectively. Remarkably, all four algorithms achieved perfect precision scores, underscoring their effectiveness in identifying outliers within the dataset.

Within the scope of this paper, we extend our previous works [6,26,27] by conducting a comprehensive overall assessment of all novelty detection and outlier detection algorithms presented in [6,26,27], using the same segment of the HuMIdb dataset. This evaluation aims to pinpoint the most effective algorithms for integration into the proposed risk-based adaptive user authentication mechanism. In the following section, we thoroughly present the following: (i) the data pre-processing and data normalization processes, (ii) the hyperparameter tuning, the training and the testing processes that we followed, and finally (iii) the overall performance evaluation of the detection algorithms.

### 3.1. Data Pre-Processing and Normalization

For training and testing the detection algorithms for the proposed mechanism, we used a new generated dataset derived from the HuMIdb dataset [28,29,41]. This new generated dataset will henceforth be referred to as the HuMIdb dataset for the rest of this paper. In particular, this new dataset consists of: (i) the data related to the user000, who was considered as the normal one, and (ii) the data related to the user001, who was considered as the malicious one.

In principle, before deploying the detection algorithms, dataset preparation is crucial. The preparation of the dataset includes (a) data pre-processing and (b) data normalization [42]. 

Although the pre-processing phase usually involves the removal of unnecessary features and the conversion of the nominal values of the categorical features to numeric values, in our case, there were no unnecessary features which were required to be removed and the values of all features were already numeric.

Afterwards, the focus was on data normalization that was crucial for ensuring that no single feature disproportionately influenced the outcome due to its scale. This was achieved through min-max normalization [43,44], scaling all feature values to a [0.0, 1.0] range, and thus maintaining uniformity across the dataset for effective algorithm training and testing. 

The following equation describes the normalization process that we used:

(1) $$z=(x-x_{min})/(x_{max}-x_{min})$$

where “z” represents the normalized value after scaling, “x” is the original value before scaling, and “xmax” and “xmin” are the maximum and minimum values of the feature, respectively.

### 3.2. Hyperparameter Tuning, Training and Testing Processes

**Figure 2:**
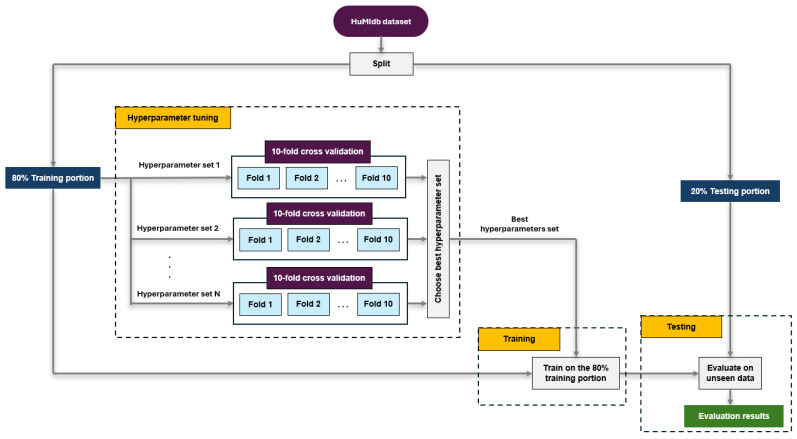
Hyperparameter tuning, training, and testing processes for each detection algorithm.

The novelty detection and outlier detection algorithms undergo the following processes: (i) hyperparameter tuning, (ii) training, and (iii) testing. Firstly, the HuMIdb dataset is divided into an 80% training portion and a 20% testing portion, as shown in Figure 2. Subsequently, the 80% training portion is used to tune the hyperparameters of the algorithm, and for that purpose, various sets of hyperparameter combinations are created. For each set of hyperparameters, a ten-fold cross-validation method is used to evaluate the performance of the algorithm on the specific set of hyperparameters, as shown in Figure 2. In particular, the performance metrics of each algorithm, using a specific set of hyperparameters, are calculated by averaging the results of the ten folds. Based on the calculated performance metrics of all sets of hyperparameters for the algorithm, the best performing set of hyperparameters for each algorithm is selected. The chosen best hyperparameters sets for each algorithm are presented in Table 1.

In the next step, each algorithm, along with its selected best hyperparameters set, is trained on the 80% training portion, as shown in Figure 2. Afterwards, each trained algorithm is tested to evaluate its performance on unseen data. In this case, the 20% testing portion played the role of the unseen data. In this way, it is possible to reduce the possibility of overfitting and get representative performance results on new and unseen data, which is critical for our application [42,45,46].

For our experiments, we utilized Python 3.9.7, incorporating Scikit-Learn library for general machine learning tasks and PyOD library for novelty detection and outlier detection. We crafted a Python script leveraging specific functions from these libraries to facilitate the hyperparameter tuning, the training, and the testing/evaluation [45,46] of all seven novelty detection algorithms and four outlier detection algorithms, ensuring a systematic approach to our experimental analysis.

### 3.3. Detection Algorithms Performance Evaluation

**Figure 3:**
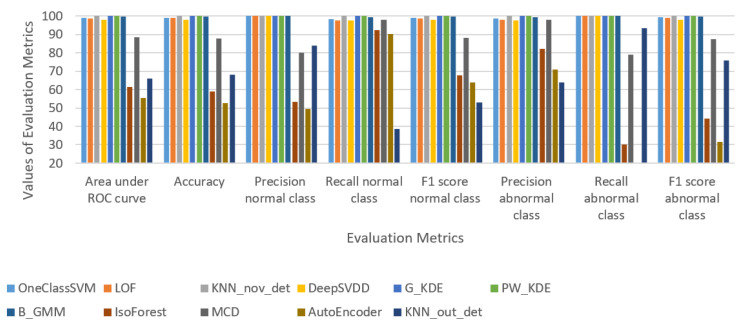
Evaluation results for detection algorithms for the HuMIdb dataset.

Figure 3 and Table 2 present the evaluation results of the seven novelty detection and four outlier detection algorithms tested in the 20% testing portion in terms of the following metrics [46]: (i) Area under ROC Curve, (ii) Accuracy, (iii) Precision on normal class, (iv) Recall on normal class, (v) F1 score on normal class, (vi) Precision on abnormal class samples, (vii) Recall on abnormal class, and (viii) F1 score on abnormal class.

Among novelty detection algorithms, B_GMM, G_KDE, KNN_nov_det, and PW_KDE stand out with exceptional performance, each achieving almost perfect scores across all metrics, including Area under ROC curve, accuracy, and F1 scores for both normal and abnormal classes. These results indicate a strong ability to correctly classify both inlier and outlier data points. However, perfect scores in evaluation results of G_KDE, KNN_nov_det, and PW_KDE may indicate overfitting, and thus, they can raise concerns about their ability to generalize well to new unseen data. This may be due to the fact that G_KDE, KNN_nov_det, and PW_KDE are too closely tailored to the specific dataset.

OneClassSVM and LOF also perform admirably, with OneClassSVM achieving a high Area under ROC curve of 99.1% and accuracy of 99.2%, and LOF slightly behind with an Area under ROC curve of 98.78%. Both exhibit near-perfect precision, recall, and F1 scores for both classes.

On the other hand, in the realm of outlier detection algorithms, the results are more varied. IsoForest and AutoEncoder present moderate to low performance, with IsoForest showing moderate precision, but lower scores in other metrics, and AutoEncoder significantly underperforming in most metrics. KNN_out_det has better performance than IsoForest and AutoEncoder, particularly in precision and F1 score, yet it does not reach the high performance of the top novelty detection algorithms.

Therefore, considering the overall performance and potential issues such as overfitting and lack of generalization, the top three algorithms from the set of 11 evaluated can be identified as follows:

1. B_GMM: demonstrates the highest performance with an Area under ROC curve of 99.6 and accuracy of 99.7, alongside perfect scores in other metrics.
2. OneClassSVM: exhibits robust performance with high scores in Area under ROC curve (99.1) and accuracy (99.2), as well as excellent precision, recall, and F1 scores.
3. LOF: delivers strong results with a slightly lower Area under ROC curve than OneClassSVM (98.8) but maintains high accuracy (98.9) and exceptional precision, recall, and F1 scores.

These three novelty detection algorithms, particularly B_GMM, are the most promising for integration into the proposed risk-based adaptive user authentication mechanism, demonstrating a balance of high performance and likely generalizability to new data.

## 4. Functional Description of the Proposed Risk-Based Adaptive User Authentication Mechanism

**Figure 4:**
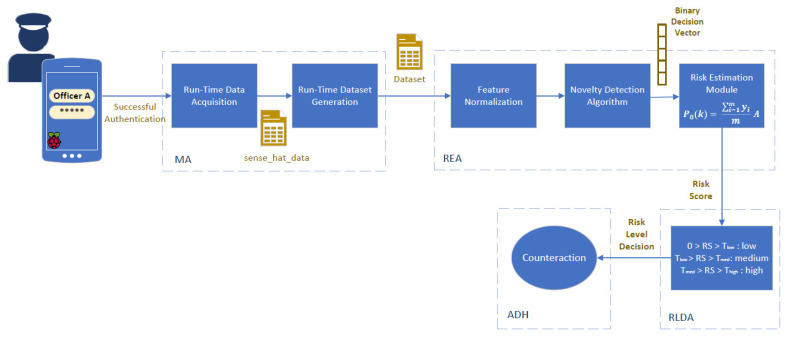
Functional diagram of the proposed risk-based adaptive user authentication mechanism.

The functional diagram, depicted in Figure 4, illustrates the operational workflow of the proposed risk-based adaptive user authentication mechanism, providing details of the specific functionalities of the components of the proposed mechanism. Upon booting the Raspberry Pi, the mechanism directly prompts the Officer to authenticate by selecting their username and entering their password.

**Figure 5:**
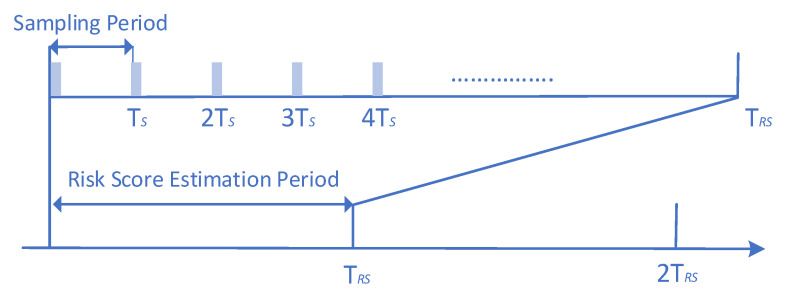
Illustration of the sampling period *Ts* and the Risk Score (RS) estimation period *TRS*.

Following the successful initial Officer authentication step, the Officer gains access to the device, and the Monitoring Agent (ΜA) component starts running. It operates silently in the background, ensuring that monitoring does not interrupt the use of the device. The Run-Time Data Acquisition block inside the MA component is responsible for collecting data related to the Officer’s activity and context, for every sampling period *Ts* (i.e., an illustration of the period *Ts* is given in Figure 5).

Then, for every risk score estimation period *T_RS_* (i.e., an illustration of the period *T_RS_* is given in Figure 5), the collected data from the Run-Time Data Acquisition block are compiled into a dataset by the Run-Time Dataset Generation block, as shown in Figure 4, and the generated dataset is indicated by the file icon labeled “Dataset”. This “Dataset” is forwarded to REA component where it undergoes Feature Normalization to ensure that all input features contribute equally to the subsequent analysis. The normalized data is then fed into the Novelty Detection Algorithm block, which applies a novelty detection algorithm to the normalized data to classify each entry as legitimate or malicious. In particular, the Novelty Detection Algorithm block applies one of the best novelty detection algorithms, which were identified as the most efficient ones in Section 3, namely OneClassSVM, LOF, and B_GMM. The outcome of the novelty detection algorithm is a value of 0 (indicating a normal user) or 1 (indicating a suspicious user) to every data point (i.e., entry), producing a “Binary Decision Vector,” whose length matches the number of data entries analyzed and is denoted as *yϵR^m x 1^*. This vector is then the input into the Risk Estimation Module, as illustrated in Figure 4, which computes the “Risk Score” (i.e, P_0_(*k*) ϵ [0, 1]) for time intervals such as *k* (e.g., *T_RS_*, 2*T_RS_)* according to the following equation [47,48,49,50]:

(2) $$P_{0}\left(k\right)=\frac{\sum_{i=1}^{m}y_{i}}{m}A$$

In this context, A symbolizes the accuracy of the novelty detection algorithm, defined by the formula:

(3) $$A=\frac{TP+TN}{TP+TN+FP+FN}$$

where:

- TP (True Positive) represents the count of correctly identified suspicious users.
- TN (True Negative) signifies the count of correctly identified normal users.
- FP (False Positive) indicates the count of normal users incorrectly labeled as suspicious.
- FN (False Negative) represents the count of suspicious users incorrectly labeled as normal.

The computed “Risk Score” is then forwarded to the Risk Level Decision Agent (RLDA) component, as also depicted in Figure 4, which takes the “Risk Level Decision” based on predefined risk thresholds (i.e., a risk score above T_high_ is considered high, between T_low_ and T_med_ is medium, and below T_low_ is low). Based on the risk level determined by the RLDA, the Authentication Decision Handler (ADH) displays a warning message on the screen, prompting additional authentication steps (i.e., re-authentication) if the risk score is medium, or device lock if the risk score is high. 

## 5. Prototype Implementation of the Proposed Risk-Based Adaptive User Authentication Mechanism

### Implementation Overview

We developed the proposed mechanism as an application on a Raspberry Pi 4 Model B device. The Raspberry Pi 4 Model B is a compact and powerful single-board computer that is popular for a variety of projects, from learning programming to home automation and industrial applications. It features a quad-core ARM Cortex-A72 processor, supports up to 8 GB of RAM, and offers full-throughput gigabit Ethernet, dual-band wireless networking, and Bluetooth 5.0. It also includes two USB 3.0 ports, two USB 2.0 ports, two micro-HDMI ports capable of 4 K video output, a 3.5 mm audio jack, and a standard 40-pin GPIO header for hardware projects. Regarding the implementation of the components of the proposed risk-based adaptive user authentication mechanism as an application on the Raspberry Pi 4 device, we crafted scripts in Python 3.9.7. 

**Figure 6:**
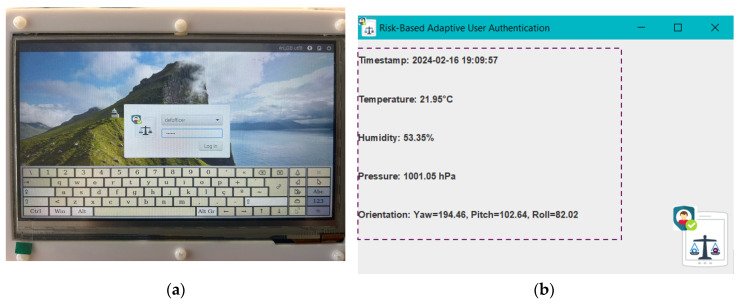
User interface: (**a**) Initial Officer authentication; (**b**) Monitoring agent component.

Upon booting the Raspberry Pi, the mechanism directly prompts the Officer to choose their username and enter their password (using a virtual keyboard), as shown in Figure 6a. This initial Officer authentication relies on LDAP (Lightweight Directory Access Protocol), and, for our implementation purposes, the LDAP authentication was enabled by restarting the *nslcd* and *nscd* services and configuring them to start at boot. LDAP is a protocol that facilitates the management and access of directory information over an IP network. In our implementation, the LDAP client runs on the Raspberry Pi 4 device to manage the connection and communication with the LDAP server for Officer authentication. It handles the submission of Officers’ credentials (i.e., username and password) for verification against the LDAP directory, where Officer’s credentials are stored. If the username and password match the credentials stored in the directory, the Officer is authenticated and granted access to the device. In our implementation, the LDAP server runs on an Ubuntu machine.

**Figure 7:**
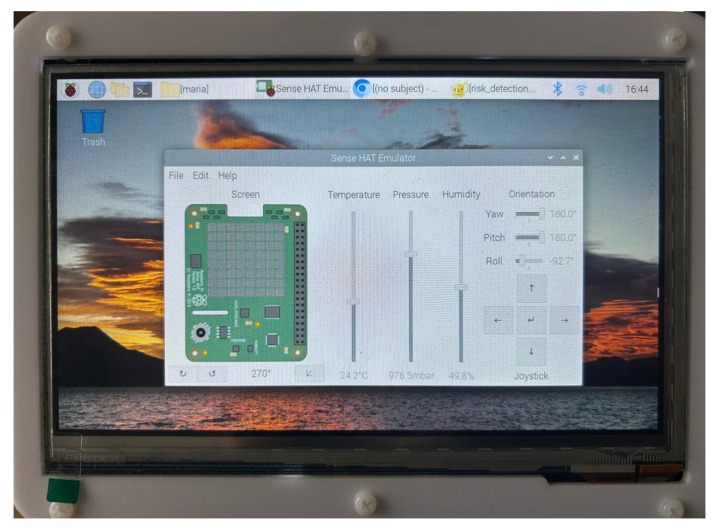
Sense HAT emulator.

Following the successful initial Officer authentication step, the Officer gains access at the device and, behind the scenes and without interrupting the Officer’s normal activities and missions, the MA starts to collect data related to the Officer’s activity and context. The collected data are printed in the user’s interface, as shown in Figure 6b. For demonstration purposes, we utilized the Sense HAT board [51] to generate the data related to the Officer’s activity and context. The Sense HAT is an add-on board for Raspberry Pi that includes a suite of sensors providing data on device orientation (through a gyroscope, accelerometer, and magnetometer), pressure, humidity, and temperature, as well as an 8 × 8 LED matrix for display purposes and a small joystick for input, as shown in Figure 7. The data generated by the Sense HAT and related to the Officer’s activity and context are collected by the MA component and used by the REA component to estimate the risk score, which is essential for the RLDA component to take risk level decisions based on the predefined risk thresholds, as described in Section 4. In the REA component, we implemented the best novelty detection algorithms (i.e., OneClassSVM, LOF, and B_GMM), identified in Section 3, as Python scripts in the Novelty Detection Algorithm block. Based on their implementation, their performance evaluation is carried out in Section 6 to identify the most suitable novelty detection algorithm for the proposed mechanism running as an application on the Raspberry Pi 4 device. The outcome of the most suitable novelty detection algorithm produces the input for the Risk Estimation Module in the REA component to calculate the risk score.

**Figure 8:**
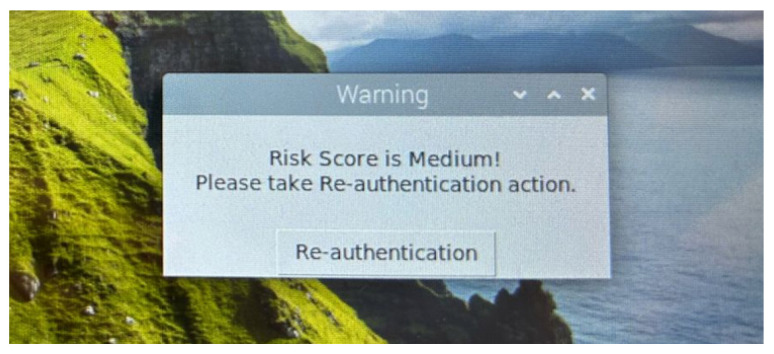
User interface: alert for medium risk score.

**Figure 9:**
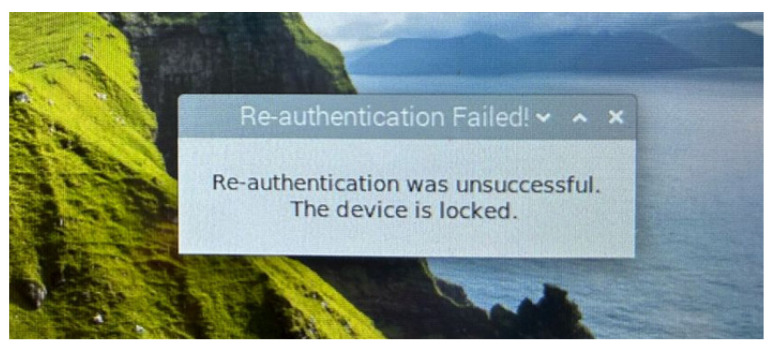
User interface: unsuccessful re-authentication.

**Figure 10:**
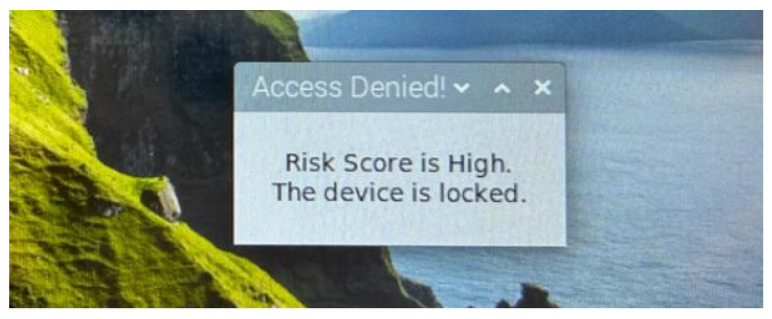
User interface: alert for high risk score.

When the risk score is determined as low by the RLDA, the Officer remains signed in. However, when the risk score is medium, a warning message appears on the screen, requesting the Officer to provide additional authentication information (i.e., re-authentication/re-enter password), as shown in Figure 8. If the re-authentication is successful, the Officer is allowed to remain signed in, otherwise the device is locked and a “Re-authentication Failed!” message appears in the user interface, as shown in Figure 9. Similarly, when the risk score is high, the device is locked again, and an “Access Denied!” message appears in the user interface, as shown in Figure 10.

## 6. Performance Evaluation

### 6.1. Experimental Setup

To evaluate the detection performance and the risk estimation overhead of the proposed mechanism running on a Raspberry Pi 4 Model B device, we conducted several experiments, using the Sense HAT board which was attached to the Raspberry Pi. The Sense HAT board was configured to generate the following types of data: device orientation (i.e., yaw, pitch, roll), pressure, humidity, and temperature. 

**Figure 11:**
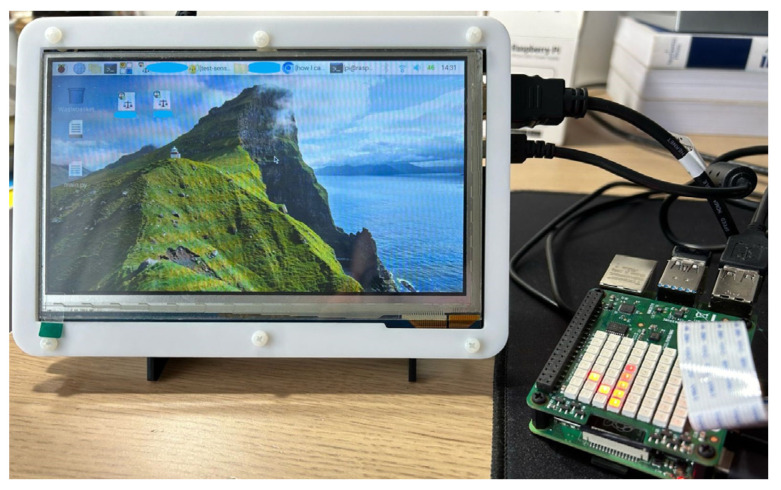
Experimental setup.

Figure 11 shows our experimental setup, consisting of the following: (i) the Raspberry Pi 4 Model B device where the proposed authentication mechanism runs, (ii) the 7-inch HDMI LCD (H) connected to the Raspberry Pi device where the Officer is prompted to choose their username and enter their password for authentication purposes, and (iii) the Sense HAT board connected on top of the Raspberry Pi device for generation of data related to the Officer’s activity (i.e., device orientation) and Officer’s context (i.e., pressure, humidity, and temperature). 

### 6.2. Detection Performance

In Section 3, we found that the three novelty detection algorithms, namely OneClassSVM, LOF, and B_GMM, are the most suitable for the proposed risk-based adaptive user authentication mechanism, demonstrating a balance of high performance and likely generalizability to new data. Therefore, after fine-tuning these algorithms based on the best hyperparameters identified in Section 3 and presented in Table 3, we developed them, as mentioned in Section 5, into the Novelty Detection Algorithm block of the REA component in the proposed mechanism. We evaluated their performance in real-time on the Raspberry Pi 4 Mode l B device. The evaluation focused on the following several key metrics: Area under the ROC curve, accuracy, precision on both normal and abnormal classes, recall on both normal and abnormal classes, and F1 score on both normal and abnormal classes.

**Figure 12:**
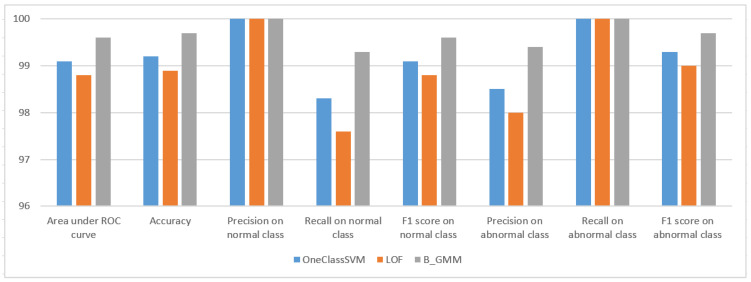
Evaluation results for the novelty detection algorithms in real-time on the Raspberry Pi device.

Our results demonstrated that the OneClassSVM novelty detection algorithm exhibited robust performance with a 99.2 Area under the ROC curve and 99.2 accuracy, as shown in Figure 12. It demonstrated perfect precision for normal class detection and very high recall and F1 score for both normal and abnormal classes, indicating a strong ability to identify legitimate and malicious users accurately.

In addition, LOF also demonstrated high effectiveness with a 98.8 Area under the ROC curve and 98.9 accuracy, as shown in Figure 12. Similar to OneClassSVM, it achieved perfect precision in identifying normal class entries and high scores across recall and F1 metrics, though it slightly lagged behind OneClassSVM.

Nevertheless, B_GMM outperformed both OneClassSVM and LOF novelty detection algorithms, giving the highest scores across all evaluation metrics, including a 99.8 Area under the ROC curve and 99.7 accuracy as shown in Figure 12. It maintained perfect precision in classifying normal class entries and near-perfect recall and F1 scores for both classes, positioning it as the top-performing algorithm in this evaluation.

The summary of the evaluation results for the three novelty detection algorithms when running in real-time on the Raspberry Pi 4 Model B device are presented in Table 4.

### 6.3. Risk Estimation Overhead

In the development of the proposed risk-based adaptive user authentication mechanism, the concept of risk estimation overhead emerges as a pivotal consideration. To evaluate the risk estimation overhead of the proposed mechanism running on a Raspberry Pi 4 Model B device, we conducted an empirical evaluation of its runtime performance on the Raspberry Pi 4 device. In particular, during this evaluation, our experiments were designed to measure the risk estimation overhead introduced by the Risk Estimation Agent (REA) component when operating in real-time on the Raspberry Pi 4 device and the best performing B_GMM was deployed. We defined the sampling period (TS) as 2 s and varied the risk score estimation period (TRS) across three different durations: 2, 5, and 10 min.

**Figure 13:**
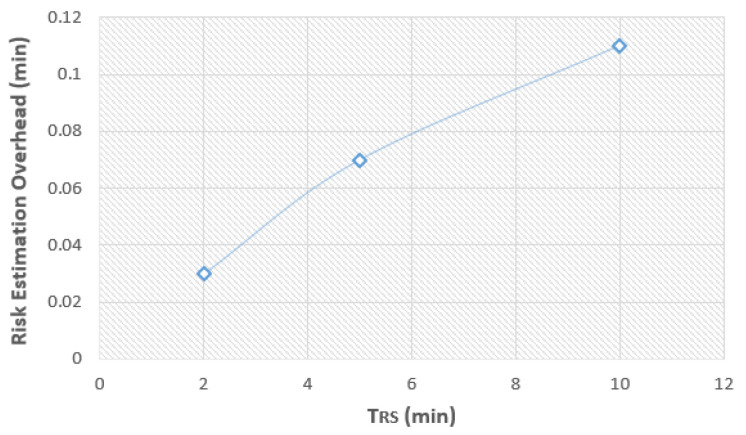
Risk estimation overhead in real time on the Raspberry Pi 4 Model B device.

Our results, presented in Table 5, indicate a near-linear relationship between the TRS and the risk estimation overhead time, as shown in Figure 13. The incremental overhead of 1.8 s for a TRS of 2 min, 4.2 s for a TRS of 5 min, and 6.6 s for a TRS of 10 min suggests that the REA component of our mechanism operates efficiently, with minimal additional latency, which is an important characteristic for real-time applications.

It is crucial to highlight that the proposed mechanism allows for adjustable risk score estimation periods (TRS), set at 2, 5, and 10 min, directly correlating with the criticality of the application. For instance, for applications requiring high-security, a 2-min TRS is preferred, offering rapid detection and response capabilities. Conversely, less sensitive applications might opt for a 10-min TRS leading to an overhead of 0.11 min for the interval of 10 min. This overhead is less compared to the incurred overhead of the 2-min TRS (5 × 0.03 = 0.15 min) when it is considered for the interval of 10 min. Nevertheless, despite the lower overhead of the 10-min TRS, detection of potential malicious activity can be delayed. This flexibility ensures that the mechanism’s vigilance is appropriately matched to the application’s security needs, optimizing resource utilization without compromising the integrity of the authentication process.

## 7. Conclusions

With the rise in EU visitor numbers, the importance of adopting innovative border control solutions, such as mobile devices for land and sea border passenger identification, is paramount to ensure both passengers’ and officers’ convenience and safety. These devices, which are expected to handle sensitive personal data, become prime targets for malicious entities aiming to exploit or steal these data. Thus, to ensure the security of such devices while maintaining the smooth conduct of border control operations, strong user authentication mechanisms are necessary. In response to this challenge, we introduced a risk-based adaptive user authentication mechanism designed specifically for mobile devices utilized in land and sea border control, with the goal of reinforcing device security seamlessly alongside user convenience (i.e., usability). This work provides an extensive evaluation of novelty and outlier detection algorithms, and ultimately identifies OneClassSVM, LOF, and B_GMM as the most efficient for risk estimation for the proposed mechanism. Additionally, in this work, we developed the proposed risk-based adaptive user authentication mechanism as an application on a Raspberry Pi 4 Model B device, which serves as the mobile device for passenger identification. The detection performance of the three leading novelty detection algorithms (i.e., OneClassSVM, LOF, and B_GMM), as identified in Section 3, was scrutinized, revealing B_GMM’s superior performance on the Raspberry Pi 4 device. Lastly, we evaluated the risk estimation overhead incurred by the proposed mechanism when integrating the high-performing B_GMM algorithm for risk estimation, demonstrating its efficient functionality with only marginal latency increase.

The implications of our findings are substantial for land and sea border control. The deployment of a risk-based adaptive user authentication mechanism can significantly enhance the security of mobile devices used at borders without compromising usability. This balance is critical to ensure that security measures do not impede the efficiency and effectiveness of border control operations. Furthermore, the mechanism’s ability to operate on cost-effective hardware like the Raspberry Pi 4 Model B highlights its practical applicability and potential for widespread adoption across various border control settings.

**Figure 14:**
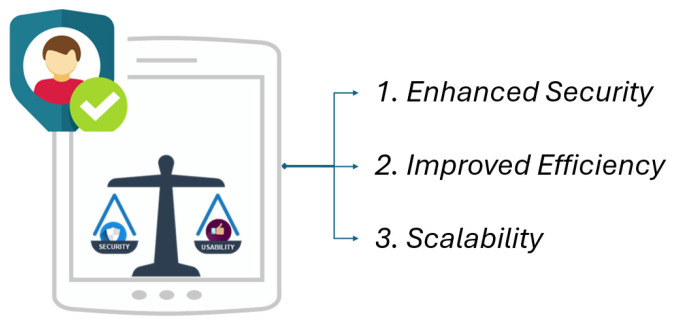
Advantages of the proposed mechanism in land and sea border control.

In particular, the potential impact on border control practices, also depicted in Figure 14, includes: (1) Enhanced Security: The proposed mechanism provides a robust solution to the security vulnerabilities of mobile devices used in border control, protecting sensitive personal data from malicious attacks. This ensures a higher standard of security and trust in border control processes; (2) Improved Efficiency: By maintaining high usability, the mechanism ensures that border control operations remain smooth and efficient, facilitating the increased flow of passengers. This operational efficiency is crucial in handling the growing volume of travelers without compromising security; (3) Scalability: The mechanism’s implementation on a cost-effective device like the Raspberry Pi 4 Model B demonstrates its scalability and potential for widespread adoption. This affordability makes it applicable to various border control agencies, enabling uniform security enhancements across different regions.

For future work, there are multiple pathways to enhance this work. Initially, it is worthwhile to combine the outcomes of multiple detection algorithms to further improve the detection performance of the proposed mechanism. Moreover, there is still a scarcity of enriched training datasets for risk-based user authentication. Therefore, we plan to create richer datasets, constructed from a wider spectrum of legitimate and malicious user behavior, and test the proposed mechanism against them. Finally, we are planning to further improve the performance of the proposed mechanism by considering a wider range of data related to the Officer’s activity and context. However, sensitive data related to the Officer’s activity (e.g., keystroke dynamics, swipe up and down etc.) and context (e.g., GPS location) may reveal the Officer’s identity, thus compromising the Officer’s privacy. Therefore, privacy-preserving mechanisms such as AnonySense [52], Medusa [53], and PEPSI [54], are essential to be considered for the protection of Officers’ privacy. The focus will be on the most lightweight privacy-preserving mechanisms for implementation and integration into the proposed mechanism running on a Raspberry Pi 4 Model B device. The target will be to identify the most efficient lightweight mechanism for real-time applications such as the risk-based adaptive user authentication.

## Figures and Tables

**Table 1:** Summary of the best hyperparameters identified for the detection algorithms.

	Algorithm	Best Hyperparameters
Novelty Detection	OneClassSVM	nu = 0.01, gamma = 0.07, kernel = rbf
	LOF	algorithm = kd_tree, contamination = auto, metric = chebyshev, n_neighbors = 20, novelty = True
	KNN_nov_det	algorithm = ball_tree, contamination = 0.00001, method = largest, metric = chebyshev, n_neighbors = 5
	DeepSVDD	contamination = 0.00001, hidden_neurons = [8,4]
	G_KDE	bandwidth = 0.1, kernel = gaussian, metric = chebyshev
	PW_KDE	bandwidth = 0.4, kernel = tophat, metric = chebyshev
	B_GMM	components = 4, covariance = full
Outlier Detection	IsoForest	behaviour = new, contamination = 0.1, n_estimators = 90
	MCD	contamination = 0.1, assume_centered = false
	AutoEncoder	contamination = 0.1, hidden_neurons = [16,8,8,16]
	KNN_out_det	algorithm = ball_tree, contamination = 0.1, method = median, metric = manhattan, n_neighbors = 20

**Table 2:** Summary of the evaluation results for the detection algorithms.

	Algorithm	Area under ROC Curve	Accuracy	Precision on Normal Class	Recall on Normal Class	F1 Score on Normal Class	Precision on Abnormal Class	Recall on Abnormal Class	F1 Score on Abnormal Class
Novelty Detection	OneClassSVM	99.14	99.20	100	98.28	99.13	98.53	100	99.26
	LOF	98.78	98.87	100	97.56	98.77	97.93	100	98.96
	KNN_nov_det	100	100	100	100	100	100	100	100
	DeepSVDD	97.86	97.87	100	97.71	97.86	97.75	100	97.88
	G_KDE	100	100	100	100	100	100	100	100
	PW_KDE	100	100	100	100	100	100	100	100
	B_GMM	99.64	99.67	100	99.28	99.64	99.38	100	99.70
Outlier Detection	IsoForest	61.33	59.08	53.4	92.40	67.68	82.16	30.27	44.24
	MCD	88.58	87.89	80.19	98.14	88.26	98	79.03	87.5
	AutoEncoder	55.37	52.83	49.53	90.39	63.99	71.00	20.35	31.63
	KNN_out_det	66.14	68.13	83.85	38.74	52.99	63.84	93.55	75.89

**Table 3:** Summary of hyperparameters for the three best performing novelty detection algorithms.

Algorithm	Hyperparameters
OneClassSVM	nu = 0.01, gamma = 0.07, kernel = rbf
LOF	algorithm = kd_tree, contamination = auto, metric = chebyshev, n_neighbors = 20, novelty = True
B_GMM	components = 4, covariance = full

**Table 4:** Summary of evaluation results for the three best performing novelty detection algorithms.

Algorithm	Area under ROC Curve	Accuracy	Precision on Normal Class	Recall on Normal Class	F1 Score on Normal Class	Precision on Abnormal Class	Recall on Abnormal Class	F1 Score on Abnormal Class
OneClassSVM	99.2	99.2	100	98.3	99.1	98.6	100	99.4
LOF	98.8	98.9	100	97.6	98.8	98	100	99
B_GMM	99.8	99.7	100	99.3	99.6	99.5	100	99.7

**Table 5:** Risk estimation overhead.

TRS (min)	Risk Estimation Overhead (min)	Risk Estimation Overhead (sec)
2	0.03	1.8
5	0.07	4.2
10	0.11	6.6

## Data Availability

Data are contained within the article.
